# Early‐Onset Wild‐Type Transthyretin Amyloidosis Polyneuropathy

**DOI:** 10.1002/mus.70387

**Published:** 2026-08-24

**Authors:** Chafic Karam, Kaylee Baker, Sami Khella, Brian Drachman, Pranav Chandrashekar, MacLean Nasrallah, Shunsuke Koga, Janice Pieretti

**Affiliations:** ^1^ Department of Neurology University of Pennsylvania Philadelphia Pennsylvania USA; ^2^ Penn Amyloidosis Center Philadelphia Pennsylvania USA; ^3^ Division of Cardiovascular Medicine, Department of Medicine University of Pennsylvania Philadelphia Pennsylvania USA; ^4^ Department of Pathology and Laboratory Medicine Hospital of the University of Pennsylvania Philadelphia Pennsylvania USA

## 1 Introduction

Wild‐type transthyretin amyloidosis (ATTRwt) results from deposition of nonmutated TTR protein, most commonly affecting the heart and tenosynovial tissues [1, 2]. Several studies have suggested a link between ATTRwt and peripheral neuropathy, with incidence ranging between 3% and 74% [3]. However, ATTRwt polyneuropathy remains controversial as affected patients are typically elderly with prevalent comorbid peripheral neuropathy, and published series rarely document TTR amyloid deposition in peripheral nerves [4, 5]. We present an early‐onset, biopsy‐proven case of ATTRwt polyneuropathy.

## 2 Case Report

A 65‐year‐old man with hypertension and paroxysmal atrial fibrillation was referred for evaluation of peripheral neuropathy. His symptoms started at the age of 52 years with numbness and tingling in the right foot spreading to the left foot within a year and gradually spread to the mid‐calves. He also described stabbing pain in the feet. He later developed difficulty with balance on uneven surfaces. A year prior to his presentation, he noticed tingling in the bilateral thumbs and index fingers. He had erectile dysfunction since age 50, but no other symptoms suggestive of dysautonomia. He reported no shortness of breath or lower extremity swelling, no back pain or claudication, and no functional limitation, cycling 30 miles per day on the weekends. He rarely drank alcohol. His mother had polyneuropathy but no cardiomyopathy. She died at 87 years of age; otherwise, the family history was noncontributory.

Neurological examination demonstrated normal muscle bulk. He had mild weakness in his abductor pollicis brevis bilaterally and could only extend the toes against gravity. Tone was normal. Muscle stretch reflexes were reduced in the upper limbs and at the knees, and absent at the ankles. Sensation was impaired in a length‐dependent fashion for pinprick, vibration, light touch, and proprioception. His neuropathy impairment score was 35 [6] (Table S1). Nerve conduction studies and electromyography showed a length‐dependent sensorimotor axonal polyneuropathy and superimposed median neuropathies at the wrists (Tables 1 and 2). Formal autonomic reflex screen was not performed.

**TABLE 1:** Nerve conduction studies.

Sensory nerves			
Nerve/Recording site	Peak latency	Conduction velocity	Amplitude
	ms	m/s	μV
R Median, Ulnar—Mid Palm			
Median Mid Palm	2.8 (≤ 2.2)	35 (≥ 50)	6.8 (≥ 50)
Ulnar Mid Palm	2.1 (≤ 2.2)	66 (≥ 50)	11.4 (≥ 12)
R Median—Index (Antidromic)			
Wrist	5.3 (≤ 3.5)	43 (≥ 50)	2.6 (≥ 20)
L Median—Index (Antidromic)			
Wrist	5.3 (≤ 3.5)	42 (≥ 50)	6.8 (≥ 20)
R Sural—Ankle			
Lower leg	NR (≤ 4.4)	NR (≥ 40)	NR (≥ 6)
R Ulnar—Digit V (Antidromic)			
Wrist	4.4 (≤ 3.1)	48 (≥ 50)	12.8 (≥ 17)
R Radial—Anatomical snuff box (Antidromic)			
Forearm	3.1 (≤ 2.9)	46 (≥ 50)	4.9 (≥ 15)

Motor nerves				
Nerve/Recording site	Distal latency	Duration	Velocity	Amplitude
	ms	ms	m/s	mV
R Median—APB				
Wrist	4.3 (≤ 4.4)	6.9		8.5 (≥ 4.0)
Elbow	10.6	8.3	49 (≥ 49)	7.7
L Median—APB				
Wrist	4.7 (≤ 4.4)	5.6		6.1 (≥ 4.0)
Elbow	11.3	6.4	41 (≥ 49)	5.1
R Ulnar—ADM				
Wrist	2.8 (≤ 3.3)	5.3		9 (≥ 6.0)
Below elbow	7.1	5.9	58 (≥ 49)	7.9
Above elbow	9.1	6.9	50	7.3
R Peroneal (Fibular)—EDB				
Ankle	NR (≤ 6.5)	NR	NR (≥ 44)	NR (≥ 2.0)
Fibula (head)				
R Peroneal (Fibular)—TA				
Fibula (head)	2.5 (≤ 6.7)	14.8		3.3 (≥ 3.0)
Popliteal fossa	4.1	14.4	56 (≥ 44)	2.9
R Tibial—AH				
Ankle	NR (≤ 5.8)	NR	NR (≥ 41)	NR (≥ 4.0)

*Note:* Normal values are in parentheses.

Abbreviations: μV, microvolt; ABP, abductor pollicis brevis; ADM, adductor digiti minimi; AH, adductor hallucis; EDB, extensor digitorum brevis; m/s, meter per second; MS, millisecond; NR, not recordable; TA, tibialis anterior.

**TABLE 2:** Needle electromyography.

Muscle	Spontaneous activity	Motor unit morphology		Amp	Poly	Recruitment	FR	Activation
	Fibs/PSW	Fasc	Dur					
R. Tibialis anterior	1+	None	Increased	Increased	25%–50%	Mod Red	Increased	Full
R. Gastrocnemius (Medial head)	1+	None	Increased	Increased	25%–50%	Mod Red	Increased	Full
R. Vastus medialis	None	None	Increased	Increased	25%–50%	Mild Red	Increased	Full
R. Tensor fasciae latae	None	None	Normal	Normal	Normal	Normal	Normal	Full
R. Iliopsoas	None	None	Normal	Normal	Normal	Normal	Normal	Full
R. Gluteus maximus	None	None	Normal	Normal	Normal	Normal	Normal	Full

Abbreviations: Amp, amplitude; Dur, duration; Fasc, fasciculation potentials; Fibs/PSW, fibrillation potentials and positive sharp waves; FR, firing rate; mid, mild; Mod, moderate; Poly, polyphasic morphology.

Laboratory testing showed normal serum and urine protein electrophoresis, with no paraprotein on immunofixation, and normal kappa and lambda free light chains. Hemoglobin A1c was 5.3% (normal < 5.7%) and vitamin B12 was 664 pg/mL (normal 200–1100 pg/mL). *TTR* gene sequencing revealed no variants. Sural nerve and gastrocnemius muscle biopsies demonstrated amyloid deposition on Congo red staining (Figure 1). Liquid chromatography–tandem mass spectrometry on Congo red–positive material from the nerve specimen identified TTR‐type amyloid. No variant TTR peptide was detected, and *TTR* sequencing identified no pathogenic variant, supporting ATTRwt. Cardiac magnetic resonance imaging (MRI) demonstrated findings highly suggestive of cardiac amyloidosis including severely increased left ventricle (LV) wall thickness with specific imaging sequences for tissue characterization demonstrating abnormal globally elevated extracellular volume (ECV) and T1 relaxation times with reverse nulling pattern which are specifically a consequence of extracellular infiltration of amyloid fibrils in the myocardium. Single photon emission computed tomography and computed tomography SPECT/CT images acquired 2 h after intravenous administration of TC‐99 m hydroxymethylene diphosphonate were strongly suggestive of transthyretin cardiac amyloidosis. NT‐proBNP was elevated at 1231 pg/mL (≤ 124 pg/mL), and high‐sensitivity troponin T was elevated at 37 ng/L (normal < 22). The patient was started on tafamidis for ATTR cardiomyopathy; vutrisiran was subsequently added following nerve biopsy results, given its potential role in ATTRwt polyneuropathy. At 2‐year follow‐up, there were no changes in neurological symptoms or signs.

**FIGURE 1:**
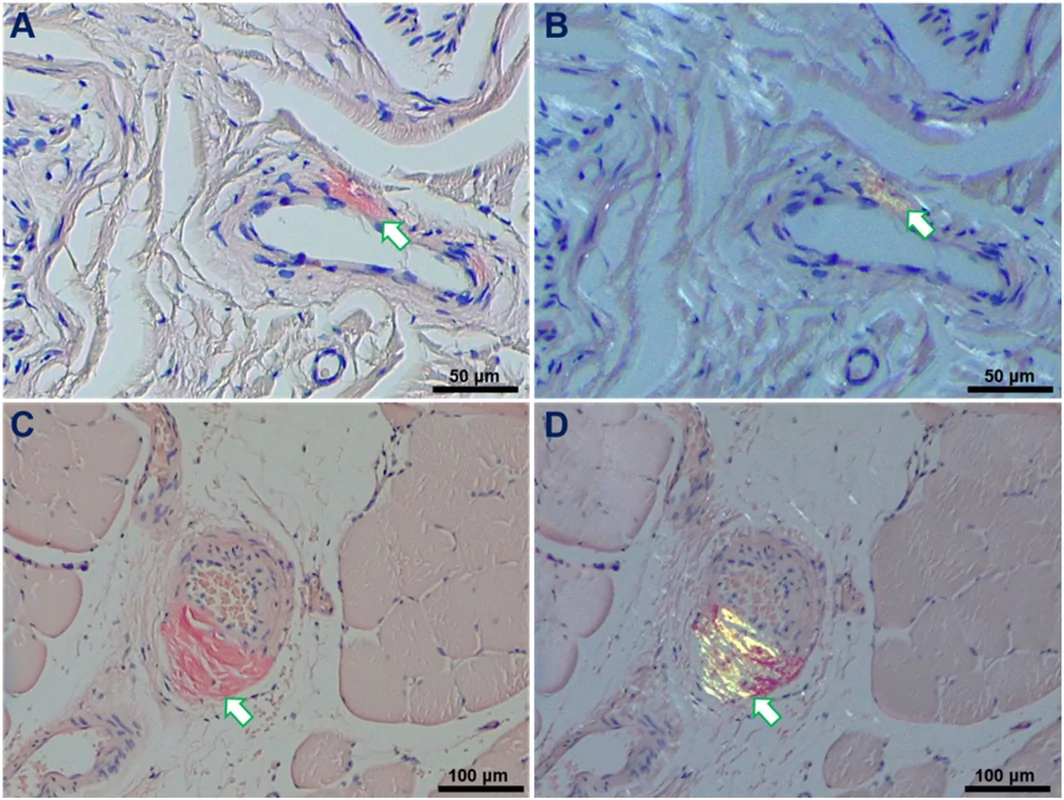
Amyloid deposition in sural nerve and gastrocnemius muscle biopsies. (A) Congo red staining of the sural nerve biopsy shows congophilic material in the wall of a small epineurial vessel (arrow). (B) The corresponding field under polarized light shows green‐yellow birefringence (arrow). (C) Congo red staining of the gastrocnemius muscle biopsy shows congophilic material in the wall of an intramuscular vessel (arrow). (D) The corresponding field under polarized light shows green‐yellow birefringence (arrow). Scale bars represent 50 μm in A and B and 100 μm in C and D.

## 3 Discussion

The patient presented here has several uncommon features. First, the age of onset is unusual for ATTRwt. ATTRwt is a disease of the elderly, with an average age of onset around 75 years [7]. Second, the fact that the initial symptoms were those of a polyneuropathy rather than carpal tunnel syndrome or the heart has been seldom described [8]. Finally, the presence of amyloid ATTRwt in the peripheral nerve has rarely been reported [9, 10]. In one report, the patient was 94 years old; in another, the mean age was 78 years. An additional report described an 84‐year‐old woman in whom amyloid was found on muscle but not nerve biopsy [11].

ATTRwt polyneuropathy remains controversial. One challenge is whether the presence of these deposits of wild‐type TTR amyloid in the nerve is the cause of the neuropathy, especially when the incidence of ATTRwt increases with age. Some studies suggest that 25% of people over the age of 85 years have wild‐type TTR deposition on autopsy, and the incidence of neuropathy increases significantly with age [4, 12]. However, the patient reported here underwent an extensive work‐up to exclude other causes, making ATTRwt the likely etiology. The reasons that some individuals develop ATTRwt are unclear. Age and male sex are considered major risk factors [13]. A recent study suggested an association with greater occupational upper‐body mechanical stress [14]. Notably, while ATTRwt is a disease of the elderly, symptom onset may precede diagnosis by over a decade [2].

Nerve biopsy is not routinely useful in evaluating chronic slowly progressive polyneuropathy, and we do not recommend it; however, the coexistence of cardiac ATTRwt in our patient justified its use to identify ATTRwt as the cause of the PN. TTR silencers may be superior to stabilizers in ATTRv PN and possibly ATTRwt PN, though combination therapy has not been shown to be superior to monotherapy [15, 16, 17].

This case highlights that ATTRwt should be considered in the differential diagnosis of idiopathic axonal polyneuropathy with known cardiac ATTRwt, and that TTR silencers may warrant consideration, though evidence remains insufficient to recommend routine biopsy or to alter standard treatment protocols.

## Author Contributions

**Brian Drachman:** writing – review and editing, data curation. **Chafic Karam:** data curation, writing – review and editing. **Janice Pieretti:** writing – review and editing, data curation. **Sami Khella:** data curation, writing – review and editing. **Kaylee Baker:** writing – review and editing, data curation. **Pranav Chandrashekar:** data curation, writing – review and editing. **Shunsuke Koga:** data curation, writing – review and editing. **MacLean Nasrallah:** writing – review and editing, data curation.

## Ethics Statement

We confirm that we have read the Journal's position on issues involved in ethical publication and affirm that this report is consistent with those guidelines.

## Conflicts of Interest

Chafic Karam is a consultant for Alexion, Alnylam, Amgen, Amicus, Annexon, Applied Therapeutics, Argenx, Astra Zeneca, Catalyst, Corino, Biogen, CSL Behring, Genentech, Immunovant, Intellia, Ionis, J&J, Medison, Neuroderm, Novo Nordisk, Nuvig, Octapharma, Pfizer, Sanofi, UCB, Takeda, Vertex, and Zai Lab. Sami Khella is a consultant to Alexion, Alnylam, AstraZeneca, Bridge Bio, Ionis, Pfizer; Brian Drachman is a consultant for Alnylam, Bridge Bio and Pfizer. The other authors declare no conflicts of interest.

## Supporting information


**Table S1:** Neuropathy impairment score.

## Data Availability

The data that support the findings of this study are available from the corresponding author upon reasonable request.
