# Supplementary material for: Early‐Onset Wild‐Type Transthyretin Amyloidosis Polyneuropathy
Source: Muscle Nerve. 2026 Aug 24;74(5):1471–5. doi: 10.1002/mus.70387 (PMC13644886; doi:10.1002/mus.70387)
Supplement: Supplementary file 1 [file MUS-74-1471-s001.docx]

Supplementary Table. Neuropathy Impairment score

| NIS Domain & Individual Assessment Sites | Patient Score |
| --- | --- |
| I. Cranial Nerves (Bilateral) | 0 |
| * Facial Weakness, Ocular Movements, Tongue, etc. | 0 |
| II. Muscle Weakness (Bilateral) | 6 |
| * Hand Finger Intrinsic (APB) | 2 |
| * Toe Extension (EHL) | 4 |
| * All other tested muscle groups | 0 |
| III. Reflex Loss (Bilateral) | 12 |
| * Biceps Brachii | 2 |
| * Brachioradialis | 2 |
| * Triceps | 2 |
| * Quadriceps Femoris | 2 |
| * Triceps Surae | 4 |
| IV. Sensation Loss (Bilateral) | 17 |
| * Touch-Pressure | 4 |
| * Pinprick Sensation | 4 |
| * Vibration Sensation | 6 |
| * Joint Position Sense | 3 |
| TOTAL NIS COMPOSITE SCORE | 35 |
